# Trade-Offs in Male Display Activity with Lek Size

**DOI:** 10.1371/journal.pone.0162943

**Published:** 2016-09-28

**Authors:** César Cestari, Bette A. Loiselle, Marco Aurélio Pizo

**Affiliations:** 1 Departamento de Zoologia, Universidade Estadual Paulista ‘‘Julio de Mesquita Filho” (Unesp), Avenida 24A, 1515, Bela Vista, Rio Claro, SP, CEP 13506-900, Brazil; 2 Department of Wildlife Ecology and Conservation and Center for Latin American Studies, P.O. Box 110430, University of Florida, Gainesville, FL, 32611, United States of America; Universita degli Studi di Sassari, ITALY

## Abstract

In lek mating systems, males aggregate and defend arenas where they display for females; females select and mate with a male and then solely raise their offspring. Generally, female visits and copulations increase and reproductive variance in male mating success declines with lek size. Here we investigate how male display effort changes across a gradient in lek size. We expect male display effort, an energetically expensive activity, will increase with lek size and male rank due to changes in breeding opportunities and competition among males. We test the interaction of male rank and lek size on display effort using the white-bearded manakin, *Manacus manacus* (Aves: Pipridae), a well-studied species with a wide geographic distribution in the new world tropics. We used mini-video recorders to simultaneously capture female visits and display behaviors of 41 males distributed over 10 leks. We found that overall display effort increased disproportionately with lek size due to males of both high and low ranks increasing their display effort at larger leks. Our results suggest that increased breeding opportunities and intrasexual competition at larger leks result in males of different ranks investing similarly in increased display effort in order to attract females.

## Introduction

In lekking species, sexual selection is hypothesized to drive the evolution of motor patterns and morphological traits of males as they perform acrobatic displays and intensely compete with each other to mate with females [1]. Mechanical and vocal sounds emitted by males essentially trigger a battle for courtship success (female visits) among competing males [2, 3]. As lek size gets larger (i.e., more males), mating opportunities likely increase as female visits are more frequent at larger leks [1, 3, 4]. Indeed, reproductive skew, a measure of variance in male mating success, declines with more males present on a lek indicating that relatively more males are successful breeders [5]. Nonetheless, mating success in most lekking species is skewed in favor of a few males (hereafter called high ranking males), which after detailed inspection by females, exhibit the preferred traits and display performances [1, 6, 7, 8]. Less attractive, or low ranking males, generally act as opportunists and obtain few or no mates at all [6].

Insights on how mating success varies among males of different ranks as a function of lek size is essential to understanding lek evolution [5, 9, 10, 11]. Overall mating success is expected to increase with larger lek sizes due to female preferences, but for high ranking males, this increase is predicted to occur only up to a certain lek size [9, 10, 11]. Beyond this optimal lek size, relative mating success is predicted to decline for high ranking males as they lose their ability to monopolize visiting females. In contrast, low ranking males may achieve greater mating success at larger lek sizes, and thus, optimal lek size is predicted to be greater for these individuals when compared to alpha males. The model’s predictions of varying optimal lek size for males of different ranks, however, appears to be dependent on how the relative competitive differences vary among males as a function of lek size [11].

**Fig 1:**
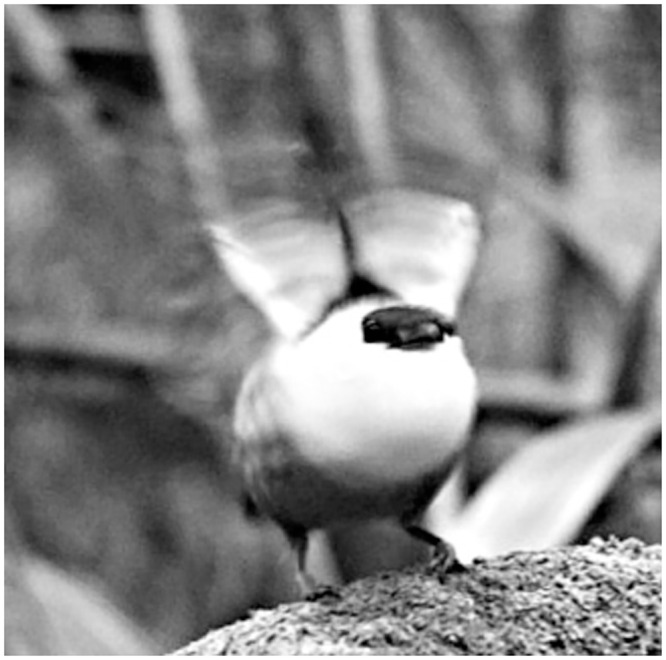
The “rolled snap” display of white-bearded manakin (*Manacus manacus*) males used to attract females to display arenas.

In the present paper, we investigate 1) how overall display effort varies with lek size and 2) how average male behaviors change with lek size as a function of their rank. We ultimately aim to understand how males deal with potential trade-offs between attracting females and increased male-male competition in leks of different sizes. We studied the white-bearded manakin (*Manacus manacus*) whose behavior, display maneuvers, vocal and mechanical sounds are broadly described in the literature [12, 13, 14, 15]. Leks of *M*. *manacus* are highly variable in size ranging from 2 to 70 individual male display arenas, but typically contain about 4–10 males, separated from each other by 0.9 to 82 m [12, 16, 17, 18]. *Manacus* males use a “rolled snap” to attract females to lek areas and display arenas [12, 15, 19]. The “rolled snap” is a loud, mechanical sound that results when a perched white-bearded male raises and rapidly vibrates its wings above his back in a succession of snaps (Fig 1). During a rolled snap, *Manacus* males may snap their wings at a rate of approximately 50–60 snaps/sec and heart rates of males may reach up to 1374 beats/min suggesting a high energetic cost equivalent to that experienced by hummingbirds during hovering flights [8, 20].

**Fig 2:**
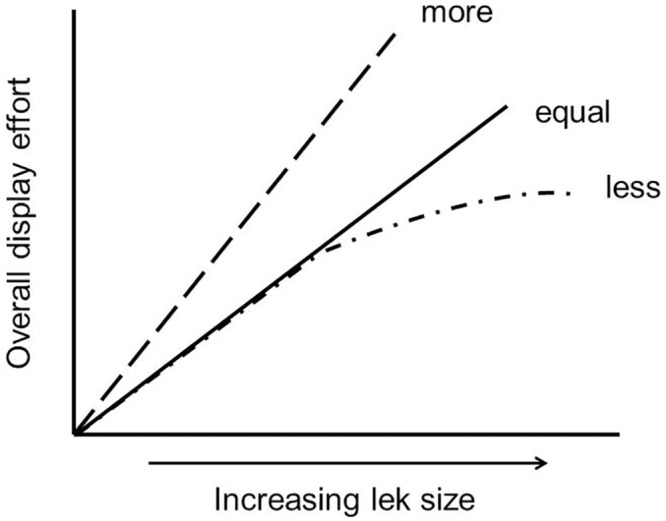
Theoretical relationship between lek size and overall display effort (i.e., display effort aggregated across all males on a lek). “More” indicates increased relative effort in display activity among males with increasing lek size, reflecting increased competition among males to attract mates. “Equal” and “less” reflect proportional or decreased display activity among males with increasing lek size, respectively.

**Fig 3:**
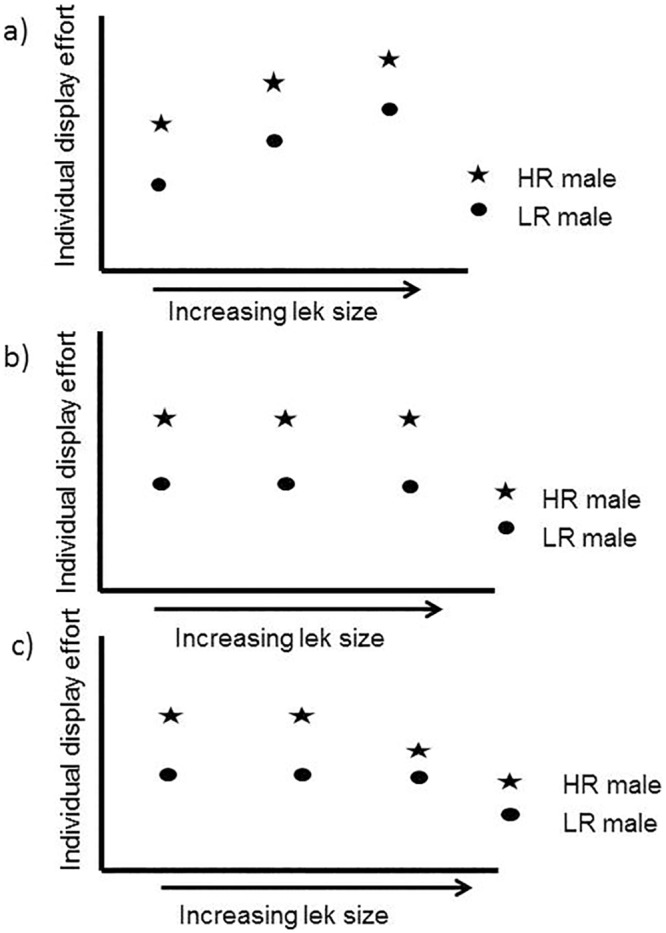
Three hypothetical relationships depicting individual display effort for high ranking (HR) and low ranking (LR) males on leks of different sizes. Overall display effort on leks (a) increases, (b) is proportional (equal) or (c) decreases with lek size. These three examples are not meant to reflect all possibilities.

With increasing lek sizes, we predict that overall display effort to attract females will increase (see Fig 2). This increase in overall display effort may occur simply because more males are present in the lek together contributing to vocal activity. Of particular interest here is the pattern of that increase in display activity with lek size. For example, if lek size increases competition among males for mates, then we might observe that average per capita effort increases disproportionately. Alternatively, there may be proportional increase in displays with more males (average per capita remains the same), or actually decreased average per capita effort in displays at highest lek sizes (Fig 2). In blue-crowned manakin males (*Lepidothrix coronata*), the average song rate of males increased disproportionately in larger leks [21]. Here, we expect a disproportionate response in overall display effort for *M*. *manacus* leks. Such a result would be influenced by different optimal lek sizes for males of different ranks, assuming that male investment in displays reflects their varying opportunities to secure mates at different lek sizes [9, 10]. However, key to this argument is an understanding of how males of different ranks adjust their behavior in leks of different sizes [11] (Fig 3). If low ranking males have relatively increased reproductive benefits on larger leks compared to smaller ones, then one might expect them to be relatively more “competitive”, as might be reflected in display effort (e.g., Fig 3a and 3c). In addition to our prediction of disproportionate increase in overall display of males according to lek size, we predict that low ranking *M*. *manacus* males will invest greater energy in displays to attract females at larger leks. Here we found that display efforts increase disproportionately with lek size and that rolled snap display efforts by high, intermediate and low ranking males are greater at larger lek sizes but not different between males.

## Material and Methods

### Study species and region

*Manacus manacus* (Pipridae) is a small 15–18 g lekking bird that inhabits the forest understory of Neotropical lowland forests from Colombia to northeast Argentina. Males exhibit black on the cap, back, wing, and tail, with gray on the rump, upper tail coverts, flanks and belly, and a white neck. Females are olive above, grayer and paler below [13]. *M*. *manacus* prefer secondary forests where they may find a high variety of small fruits to consume and small upright saplings suitable for establishment of display arenas (also called courts) [12, 15]. During the peak period of breeding, males may spend up to 90% of their daylight time in lek areas where they perform conspicuous and physically elaborate courtship displays. The period of lek activities for *M*. *manacus* may last up to seven months in tropical and subtropical regions [12, 18]. Historical records indicate that individual resident males may display on leks for up to 14 years [12, 15] and lek areas may persist for more than 40 years [22].

Lek areas of *Manacus* spp. are formed by individual display territories with ground arenas [23]. Display territories are probably established during behavioral interactions among neighboring males, including aggressive encounters that determine social hierarchy [12, 19, 15]. Individual arenas are from 0.15–0.90 m in diameter delimited by two or more saplings; resident males remove leaf litter and other debris from their arenas [12, 24, 25]. In their display territories, males utter a variety of simple calls and also perform different display elements and mechanical sounds [12, 19], including the rolled snap as the most energetically-demanding display directed to attract females to territories [8, 12, 15]. Males perform the “snap-jump” only within their arenas; this display is closely inspected by visiting females and likely very important for female mate choice decisions [12, 15, 19, 26]. In *M*. *manacus*, female visits and mating success are highly skewed and only a few males (i.e., high ranking or dominant males) achieve most of the copulations [12, 15, 19].

The study was conducted in the lowland *restinga* ecosystem of the Atlantic forest in southeast Brazil (from 24°10’S, 46°55’W to 24°33’S, 47°13’W). *Restinga* is a relatively simple vegetation composed of halophytic herbs and shrubs growing near the ocean, and more complex vegetation in lowland and foothill forests as one moves further into the continent. Climate is subtropical and humid [27]. Mean annual rainfall is 2278 mm with a rainy season from October to April, and dry season from May to September. Mean annual temperature is 21.4°C with maximum and minimum temperatures averaging 25.8°C and 19°C, respectively [28]. The most speciose plant families in the region are Myrtaceae, Leguminosae, Rubiaceae, Melastomataceae, Lauraceae, and Annonaceae [29, 30]. *M*. *manacus* is one of the most abundant manakin species in *restinga* with lek size varying from 2 to 11 arenas up to 186 m apart from each other (*n* = 14 leks) [18, 23, 25].

### Data collection

We studied 10 leks of *M*. *manacus* from September to December 2013 encompassing the peak period of lek activity [18]. Each lek had from two to seven resident males (Table 1). Straight-line distances between leks ranged from 0.33 km– 53.2 km. In each lek, we simultaneously monitored the number of rolled snaps performed by resident males, the number of female visits, and mating success (number of copulations) using Go Pro^®^ HD video cameras (one per arena). We filmed individual display areas during one morning, i.e., between the arrival of the first resident male in his territory to 0900 h (hereafter called a recording period). Cameras were camouflaged with leaves and mounted up to 5 m from arenas. Arrival times of resident males varied from 0600–0630 h and recording periods varied from 2.5 h– 3 h (Table 1). The study was observational only and was conducted following national and international guidelines. Permission to work in the field was provided by Instituto Florestal no. proc. 260108–01.144/2013.

**Table 1:** Number of resident males, recording period and ranges of a) mating success rate, b) female visits, and c) centrality of males at each of the 10 *Manacus manacus* leks.

Lek size (number of males)	Recording period (h)	Mating success (copulations/h)	Female visits (female visits/h)	Centrality (m)
2	2.81	0	0.36	11.5–29.19
2	2.61	0	0–0.77	0–13.08
3	2.91	0–0.34	0.34	4.70–37.04
3	2.96	0	0–0.34	0–16.29
3	2.76	0	0–0.72	2.72–16.19
4	2.80	0–0.71	0–11.78	4.34–74.73
5	2.50	0–0.40	0.40–1.60	7.67–20.51
6	3.00	0–0.33	0–8.70	1.29–82.47
6	2.85	0–0.70	0.35–3.18	6.03–58.3
7	2.77	0	3.61–14.44	12.5–124.99

We used the recording period within each lek to calculate rates (per hour) for the number of rolled snaps, female visits, and copulations by each male. We used the term aggregate to refer to the pooled rates of all males in a lek. We recorded lek size using the number of territorial males on a lek (Table 1). We measured the centrality of each male’s arena based on the distance from the centroid of the lek; not active arenas (during the recording period) were also used to calculate the centroid of the lek and the centrality of the active arenas. Lower values in distances from centroid indicate increased centrality of the display arena [31]. Centrality of arenas has been reported to be significantly related to the hierarchical ranking of *M*. *manacus* males, such that high ranking males (or males with higher mating success) are found to establish arenas near the centroid of leks [7, 32, 33, 34]. Copulations and centrality of territories, which is stable over the lekking season and often among years, have generally been correlated with male mating success in *Manacus* and other lekking species [7, 34]; female visits have also been shown to be correlated with copulations [35, 36]. Therefore, these three criteria were used to distinguish between the highest, intermediate, and lowest ranking males in each lek for later analysis of changes in individual display behavior based on rank. To rank males we used Principal Component Analysis (PCA) with individual males being assigned scores based on the ordination derived from values for copulation rate, female visitation rate, and centrality S1 Dataset. The first principal component explained 98.1% of the variance with eigenvalue of 829.16, while the second component explained an additional 1.88% of the variance (Table 2). Male rank within a lek (low, intermediate, high) was then assigned on a relative basis using males’ score along the first axis of the PCA. The lowest and highest scores indicated lowest and highest ranking males in each lek, respectively. Values between the lowest and highest indicate intermediate ranking males.

**Table 2:** The Principal Component Analysis results. Forty-one *M*. *manacus* males at 10 leks were used in PCA.

	PC1	PC2
Eigenvalue	829.16	15.96
Variance (%)	98.1	1.9
Centrality	0.99	-0.028
Female visitation rate	0.028	0.99
Copulation rate	-0.001	0.01

### Statistical analyses

We fitted the best relationship between rate of female visits and aggregate rolled snap rate. To examine how display effort changed in relation to lek size, we examined aggregate rolled snap rate as a function of number of males at a lek against an expected relationship using resampling techniques in R (package *boot*). The expected relationship drew males at random from the population distribution (i.e., 41 territorial *M*. *manacus* males) for leks of size 2, 3, 4, 5, 6, and 7 repeated 999 times. The mean and 95% confidence intervals of aggregate rolled snap rates for each lek size were calculated. We then compared observed values of aggregate rolled snap rate at different lek sizes to determine whether they fell below, inside, or above the expected aggregate rolled snap rates. Differences between male ranks (low, intermediate, and high) in rolled snap rate were evaluated using analysis of covariance with lek size as a covariate. The lowest, intermediate, and highest ranking males were determined using the PC scores described above. Analyses were performed in R Statistical Package, v 3.2.0 [37] and Statistix v 9.0 [38].

## Results

**Fig 4:**
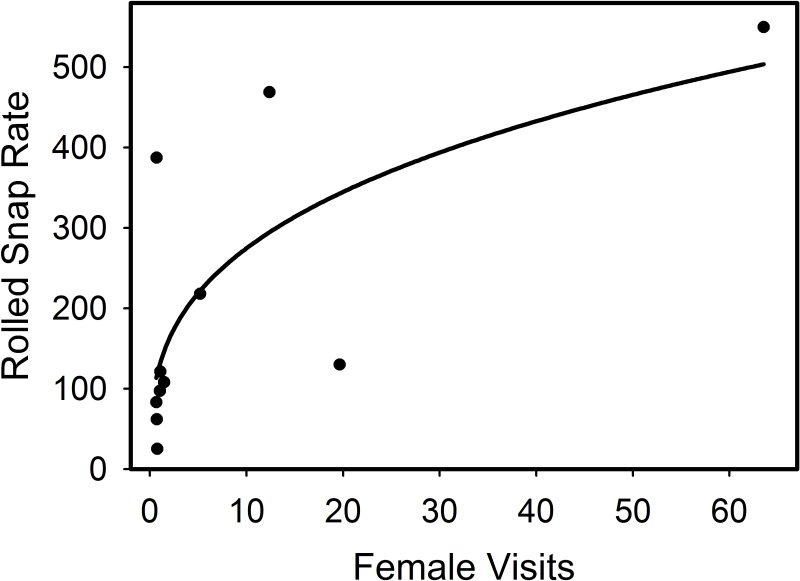
Rolled snap rate (number of rolled snaps/h, RS) increased as a function of female visitation (number/h, FV) at 10 leks of *Manacus manacus*. Function: RS = 129.16*FV^0.32^).

The number of males on 10 *M*. *manacus* leks varied from 2 to 7 (4.2 ± 1.93) (Table 1). Aggregate display effort as measured by rolled snap rates varied considerably among these leks, but as expected, we found that aggregate display effort (i.e., rolled snap rate) by males increased with female visits as measured best by a non-linear model (r^2^ = 0.48, F_1,8_ = 8.40, p = 0.018) (Fig 4). Copulations were observed on display arenas at 5 of the 10 leks (n = 5 males) which varied in size from 3 to 7 males, although the time sampled at each display arena was quite limited (Table 1).

**Fig 5:**
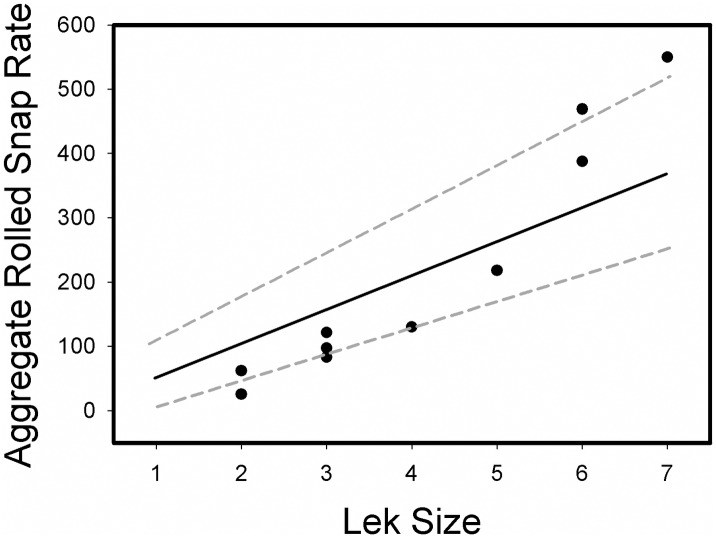
Observed aggregate rolled snap rate (#/h) at each of 10 *M*. *manacus* leks are shown as solid circles. The mean (solid line) and 95% confidence intervals (dashed lines) for aggregate rolled snap rates calculated from resampling (n = 999 times) are also shown. Values above the dashed line reflect greater display rate than expected, while those below represent relatively lower display rate than expected.

**Fig 6:**
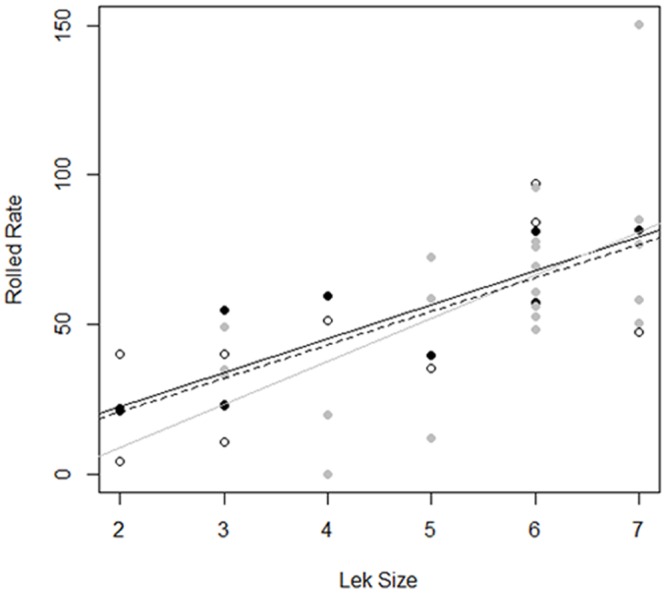
Rolled snap rate (#/h, RS) for lowest (white circles), intermediate (gray circles), and highest ranking (black circles) *M*. *manacus* males on leks of different sizes (LS). Best fit straight lines were indicated for lowest (dashed line; RS = –1.45 + 11.17*LS; r^2^ = 0.42, F = 7.56, p = 0.02), intermediate (gray line; RS = –19.82 + 14.38*LS; r^2^ = 0.34, F = 11.51, p = 0.003), and highest (black line; RS = –0.4 + 11.38*LS; r^2^ = 0.68, F = 20.52, p = 0.002) ranking males.

Aggregate rolled snap rate was found to increase disproportionately as a function of lek size (Fig 5). This disproportionate increase means that when leks contained more males, the average per capita display rate increased indicating behavioral responses by males in larger leks (see Figs 2 and 3). When we examined behaviors of lowest, intermediate, and highest ranking males, we found that display effort (i.e., rolled snap rate) of males, regardless of rank, was significantly greater in large leks (F_1,35_ = 32.35, P<0.001). In contrast, rolled snap rate did not differ as a function of male rank (F_2,35_ = 0.12, P = 0.88), or the interaction between rank and lek size (F_2,35_ = 0.21, P = 0.81) (Fig 6). These results suggest that both dominant and subordinate males changed their behaviors in similar ways on larger leks through increased individual display effort (e.g., see Figs 2 and 3a). Consequently, under apparent high competitive conditions of large leks, all males invest more energy in producing rolled snaps, a display important to attract females.

## Discussion

We found that aggregate display effort as measured by rolled snap rate increased disproportionately at larger leks of *M*. *manacus*, and males make more rolled snaps to attract more females to their arenas. These results indicate that average per capita energy expenditures by males were higher in larger leks. Our results further indicated that these higher average per capita increases in rolled snap rate were due to high, intermediate, and low ranking males increasing their display activity in larger leks. We suggest that this result can be explained by increased competition among males to secure resources (i.e., females) at larger leks, resulting in greater energy investment for potentially higher rewards [2, 5, 6].

The degree to which individual males should invest in displays is expected to vary differentially as a function of male rank and lek size to reflect varying relative benefits at different lek sizes [9,10,11]. In general, the response of reduced variance in male mating success (i.e., reproductive skew) has been shown to decrease at larger lek sizes across a variety of lekking species [5,9]. Theoretical models predict that the benefits accruing to high ranking males may accumulate faster than those of subordinate males (i.e., low and intermediate ranking males), at least up to a certain lek sizes. Beyond this optimal lek size, high ranking males may lose their ability to monopolize visiting females and reproductive skew decreases [9,10]. For instance, in ruffs (*Philomachus pugnax*) high ranking males exerted less long-distance advertising effort than lower ranking males at larger leks to potentially avoid attracting new males to the lek.

In our study, males of all ranks increased their display activity at larger leks. The differences we observed in display activity of low, intermediate, and high ranking males between small and large leks may be caused by different factors. Increased competition at large leks may cause males to invest more in displays as a result of behavioral plasticity [39]. Alternatively, higher display activity at larger leks may be a result of differential recruitment, such that males on these leks are of higher average quality and have higher display rates because they are more ‘fit’ [11, 21]. We cannot separate between these two explanations for greater display by subordinate males at larger leks. Our observations of increased aggregate display effort at larger leks suggest greater male-male competition for mates that is met by males of all ranks investing more energy in displays. With increasing lek size, high ranking males may lose their ability to monopolize females, and thus, lower-ranking males may increase their reproductive success [9,11]. Studies that predict individual benefits to males and optimal lek sizes, however, are limited [5]. Hernandez and collaborators argue that the optimal lek sizes for subordinate males may never exceed those of the highest ranking male [11], as predicted by Widemo and Owens [9], if relative competitive difference among males increases with lek size. All these models are based on the implicit assumption that males in a lek are unrelated to each other and courtship success is driven by their own success. However, we cannot discard the possibility that males in large leks are related and thus, may cooperate to attract females [40, 41, 42]; however, evidence for kin selection in manakins is mixed [40, 43].

One limitation in testing implications of models for differential male behavior as a function of lek size in our study is that data are based on behaviors of males at different leks, rather than changes in individual behavior of a given male with variation in lek size. Thus, one cannot be certain whether variation in female visits is explained by lek size or by differences in male quality that might covary with lek size. Our data are based on average behaviors of males and assume that males of large and small leks are essentially from the same pool. Given the positive association between aggregate snap rate and female visits, we suggest that male-male competition is likely driving the patterns we observed at the lek and individual male level. Controlling for male quality and other factors in captive ruffs (*P*. *pugnax*), Lank and Smith [44] found that females preferred to visit the larger of two adjacent groups of males thus corroborating the importance of average behavior among males in explaining female visits. In *M*. *manacus*, rolled snaps function to attract females [12, 19, 15] (Figs 1 and 4). Therefore, when males aggregate, rolled snap rates increase, which likely results in increased reproductive benefits to males. Indeed, Shorey also found that female visits (and number of copulations) increased at larger *M*. *manacus* leks in Trinidad [34]. Further, if male quality at larger leks is higher because of male-male competition, then reproductive benefits to females may also increase. More information regarding female mate choice processes and whether male-male competition results in males of higher quality settling in large leks are needed to better understand lek evolution in *M*. *manacus*.

## Conclusions

Lekking males may spend considerable time competing with each other for females in lek areas and, as such, the evolution of lek systems has intrigued biologists for decades [6, 45, 46]. The general increase in display effort with lek size that we observed supports the observation that rolled snap rate is likely driven by intensity of male-male competition for females. At larger lek sizes, males, regardless of status, increased display effort due to increased competition for females visiting the lek. The joint action of males performing rolled snaps in large leks of *M*. *manacus* increases overall female visits, which may benefit all males in the lek, regardless of rank. Our results suggest that males of different ranks may have similar strategies to allocate display effort when lek sizes increase. However, we recognize that results may change if the study included a greater diversity of lek sizes.

## Supporting Information

S1 Dataset Copulation rate, female visitation rate, and centrality values for each male from different lek areas. (XLSX)
